# Acute Hypoxic Respiratory Failure in Advanced Hepatocellular Carcinoma Secondary to Diffuse Pulmonary Metastases and Malignancy-Associated Hypercoagulability: A Case Report

**DOI:** 10.7759/cureus.109483

**Published:** 2026-05-23

**Authors:** Cristina Suarez Chiriboga, Besi B Sanchez, Anisha Prasad, Roxana Lazarescu

**Affiliations:** 1 Internal Medicine, Wyckoff Heights Medical Center, New York, USA; 2 Neurology, Wyckoff Heights Medical Center, New York, USA

**Keywords:** acute hypoxic respiratory failure, cancer-associated thrombosis, extrahepatic metastasis, hepatocellular carcinoma, inferior vena cava thrombus, lymphangitic carcinomatosis, malignancy-associated hypercoagulability, portal vein thrombosis, pulmonary embolism, pulmonary metastases

## Abstract

We report a 48-year-old male with newly diagnosed moderately differentiated hepatocellular carcinoma (HCC) presenting with diffuse pulmonary metastases with suspected lymphangitic involvement and extensive malignancy-associated thrombosis, including bilateral pulmonary emboli, portal vein thrombosis, and inferior vena cava thrombus. His hospital course was notable for rapid clinical deterioration with progression to acute hypoxic respiratory failure despite supportive management.

The primary lesson of this case is that diffuse bilateral pulmonary opacities with concurrent hepatic dysfunction and systemic symptoms should prompt early evaluation for metastatic malignancy and cancer-associated thromboembolism, even when infectious etiologies are initially suspected. The synergistic contribution of lymphangitic carcinomatosis and extensive venous thrombosis to refractory hypoxic respiratory failure, combined with the inability to initiate guideline-directed systemic therapy due to clinical instability precluding variceal screening, underscores the need for early prognostic assessment and timely goals-of-care discussions in advanced HCC.

## Introduction

Hepatocellular carcinoma (HCC) is the most common primary liver malignancy and remains one of the leading causes of cancer-related mortality worldwide [1,2]. Its development is closely associated with chronic liver disease and cirrhosis, and disease progression is frequently driven by delayed presentation and limited eligibility for curative therapies at advanced stages [3]. HCC is characterized by aggressive tumor biology, marked molecular heterogeneity, and a strong propensity for vascular invasion and extrahepatic dissemination.

Extrahepatic metastases most commonly involve the lungs and generally signify advanced disease with poor prognosis [4]. Pulmonary metastases classically present as multiple nodules; however, less common manifestations such as lymphangitic spread may occur and can produce severe respiratory compromise.

Malignancy-associated hypercoagulability represents an additional major contributor to morbidity and mortality in advanced cancer. Tumor-mediated activation of coagulation pathways significantly increases the risk of venous thromboembolism, including pulmonary embolism and portal vein thrombosis. Patients with HCC appear particularly susceptible to thrombotic complications because of the combined effects of vascular invasion, cirrhosis-associated hemostatic dysregulation, and tumor-driven hypercoagulability [5].

Management of advanced HCC remains challenging despite evolving systemic therapies. The combination of atezolizumab and bevacizumab has demonstrated improved survival in unresectable HCC and is currently considered first-line therapy in eligible patients [6]. Current guideline-based recommendations emphasize the importance of tumor staging, hepatic reserve, and endoscopic variceal screening prior to bevacizumab initiation because of bleeding risk [7]. Although additional systemic therapies continue to emerge, their practical applicability remains limited in patients with advanced disease and significant clinical instability [8].

Importantly, cirrhosis does not represent a naturally anticoagulated state. Contemporary understanding of cirrhosis-associated coagulation recognizes a phenomenon of “rebalanced hemostasis,” in which simultaneous reductions in procoagulant and anticoagulant pathways create a fragile equilibrium predisposing patients to both bleeding and thrombosis [9]. Pulmonary lymphangitic carcinomatosis further complicates advanced malignancy through diffuse infiltration of the pulmonary lymphatic system, producing progressive dyspnea, hypoxemia, and diffuse radiographic abnormalities that may mimic infectious or inflammatory lung disease [10].

The prognostic and therapeutic implications of lymphangitic carcinomatosis vary significantly according to the primary tumor histology. In breast cancer, lymphangitic carcinomatosis may respond to systemic therapy with prolonged survival [11]. Similar responses have been reported in colorectal carcinoma treated with anti-epidermal growth factor receptor (anti-EGFR) therapy [12] and in lung adenocarcinoma treated with targeted therapies [13]. However, reports of lymphangitic carcinomatosis associated with HCC remain limited, particularly in the setting of concurrent multifocal thrombosis and rapidly progressive hypoxic respiratory failure.

Despite the recognized associations between advanced HCC, pulmonary metastases, vascular invasion, and malignancy-associated thrombosis, the simultaneous occurrence of diffuse pulmonary metastases with suspected lymphangitic carcinomatosis, multifocal thrombosis involving multiple vascular territories, cirrhosis-associated hemostatic dysregulation, and rapidly progressive acute hypoxic respiratory failure remains insufficiently characterized in the literature. This case highlights the complex interplay between metastatic pulmonary disease, vascular invasion, systemic hypercoagulability, and therapeutic limitations in advanced HCC, underscoring the significant diagnostic and management challenges encountered in these presentations.

## Case presentation

A 48-year-old male with a past medical history of hypertension presented with progressive dyspnea, productive cough, fatigue, decreased appetite, subjective fevers, and an 18-pound unintentional weight loss over two months. He reported recent travel to the Dominican Republic, raising initial concern for infectious etiologies, including tuberculosis. He denied tobacco or illicit drug use and reported occasional alcohol consumption.

On presentation, he was hypoxic with an oxygen saturation of 90% on room air, requiring supplemental oxygen. Vital signs were notable for tachycardia and tachypnea. Physical examination revealed diffuse bilateral crackles without peripheral edema or signs of volume overload.

Initial laboratory evaluation demonstrated preserved white blood cell and platelet counts with elevated transaminases and bilirubin. A summary of laboratory trends from presentation to the end of the clinical course is provided in Table 1. Infectious workup, including COVID-19, influenza, and a respiratory viral panel, was negative.

**Table 1:** Laboratory trends from initial presentation to final hospital course. Summary of laboratory findings demonstrating progressive hepatic dysfunction and persistent hypercoagulability with preserved renal function. WBC: white blood cell count; RDW: red cell distribution width; BUN: blood urea nitrogen; AST: aspartate aminotransferase; ALT: alanine aminotransferase; GGT: gamma-glutamyl transferase; AFP: alpha-fetoprotein; HCC: hepatocellular carcinoma; INR: international normalized ratio; PTT: partial thromboplastin time

Parameter	Initial	Mid-course	Final	Reference Range	Clinical Interpretation
WBC (K/μL)	7.9	8.8	8.8	4.5-11.0	Stable, within normal range
Hemoglobin (g/dL)	17.5	16.1	15.8	13.0-17.0	Progressive decline
Hematocrit (%)	52.5	48.2	46.8	41.0-51.0	Progressive decline
Platelets (K/μL)	186	186	178	130-400	Stable
RDW (%)	18.3	18.2	18.6	10.0-14.0	Persistently elevated
Sodium (mmol/L)	137	137	138	136-145	Stable
Potassium (mmol/L)	4.8	4.2	4.4	3.5-5.1	Stable
Creatinine (mg/dL)	0.83	0.77	0.68	0.55-1.30	Preserved renal function
BUN (mg/dL)	13	13	15	7-18	Stable
Glucose (mg/dL)	103	84	95	74-106	Stable
Albumin (g/dL)	3.3	3	3	3.4-5.0	Persistently low
Total bilirubin (mg/dL)	1.4	1.8	2	0.2-1.0	Progressive increase
Direct bilirubin (mg/dL)	-	1.15	-	0.10-0.20	Elevated
AST (U/L)	240	229	297	15-37	Elevated
ALT (U/L)	181	148	184	12-78	Elevated
Alkaline phosphatase (U/L)	199	168	178	45-117	Elevated
GGT (U/L)	792	-	-	5-85	Marked elevation
AFP (ng/mL)	>200	-	-	<10	Markedly elevated
AFP-L3 fraction (%)	71.8	-	-	<10	Highly elevated, consistent with aggressive HCC biology
Lactic acid (mmol/L)	2.6	3.5	2.5	0.4-2.0	Elevated
D-dimer (ng/mL)	1158	-	-	400-500	Marked elevation
Procalcitonin (ng/mL)	0.58	-	-	0.05-0.09	Mild elevation
Phosphorus (mg/dL)	2.1	2.5	-	2.5-4.9	Initially low, corrected
Magnesium (mg/dL)	2	2.4	-	1.6-2.6	Stable
INR	1.21	-	-	0.88-1.13	Elevated
PTT (sec)	38.4	-	-	27.0-37.0	Elevated

A portable chest radiograph demonstrated diffuse bilateral nodular and confluent airspace opacities, raising concern for metastatic disease versus an infectious process (Figure 1). Given these findings, computed tomography (CT) of the chest revealed innumerable bilateral pulmonary nodules with diffuse distribution, highly suggestive of metastatic disease with findings suggestive of lymphangitic spread (Figure 2). A CT scout image further demonstrated extensive bilateral pulmonary involvement (Figure 3).

**Figure 1:**
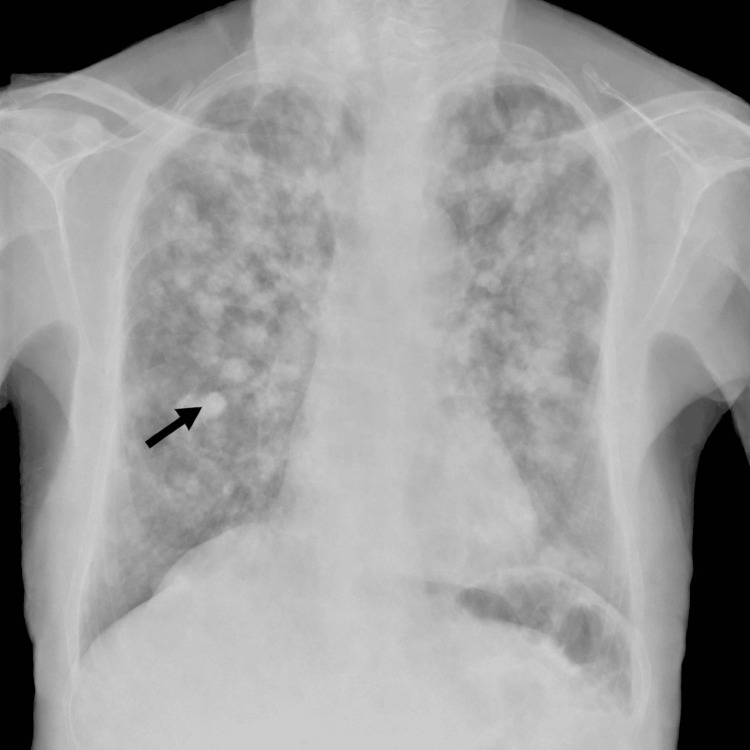
Chest X-ray demonstrating diffuse bilateral pulmonary opacities. Portable chest radiograph demonstrating diffuse bilateral nodular and confluent airspace opacities. Findings are concerning for widespread metastatic disease versus infectious etiology. The black arrow indicates a representative nodular opacity.

**Figure 2:**
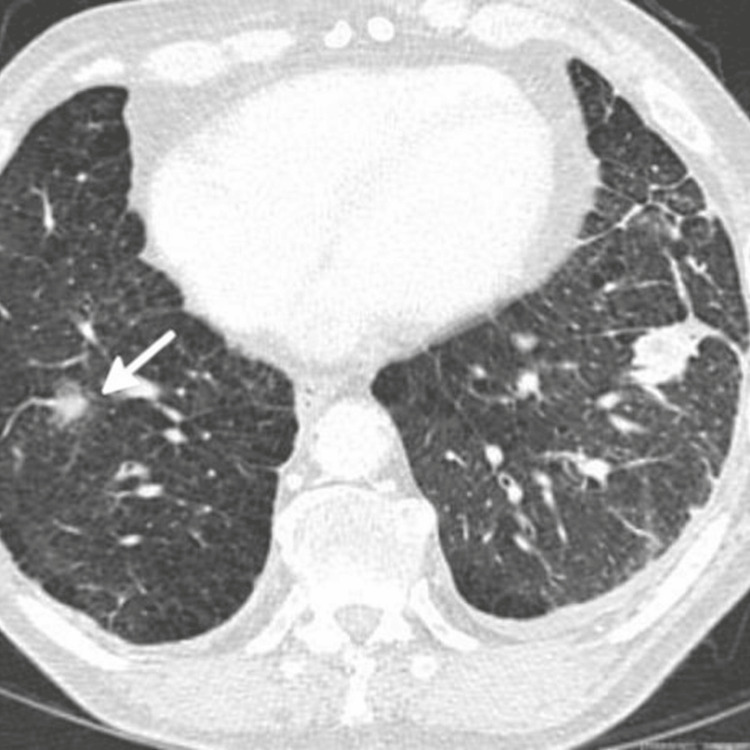
Axial CT chest demonstrating diffuse pulmonary nodules. Axial CT image of the chest showing innumerable bilateral pulmonary nodules with diffuse distribution, consistent with metastatic disease; the white arrow indicates a representative lesion.

**Figure 3:**
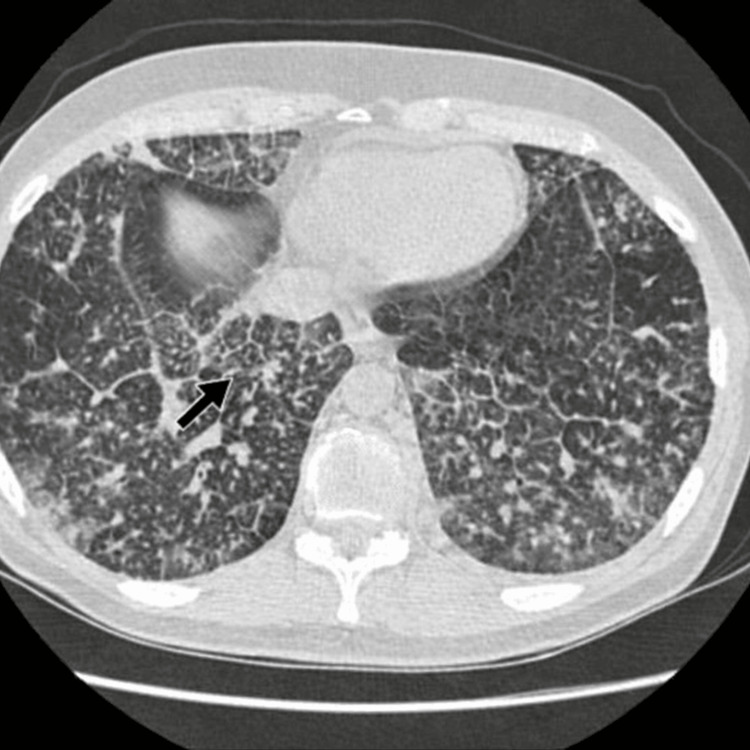
CT scout image demonstrating extensive pulmonary involvement. CT scout image demonstrating diffuse bilateral pulmonary opacities consistent with extensive metastatic involvement, correlating with advanced disease burden. The black arrow indicates a representative pulmonary opacity.

In the setting of suspected malignancy, further imaging was pursued. Abdominal ultrasound demonstrated cirrhotic liver morphology (Figure 4), and Doppler ultrasound showed absence of portal vein flow consistent with portal vein thrombosis (Figure 5). Tuberculosis workup, including QuantiFERON testing and sputum acid-fast bacilli studies, was negative.

**Figure 4:**
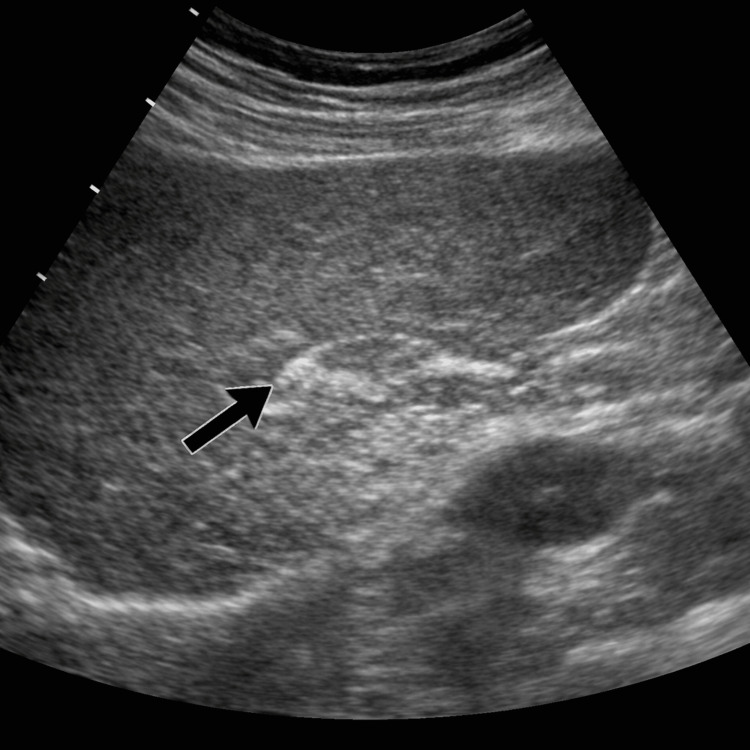
Abdominal ultrasound demonstrating cirrhotic liver morphology. Coarse echotexture and nodular contour of the liver are consistent with cirrhosis; the black arrow indicates surface irregularity.

**Figure 5:**
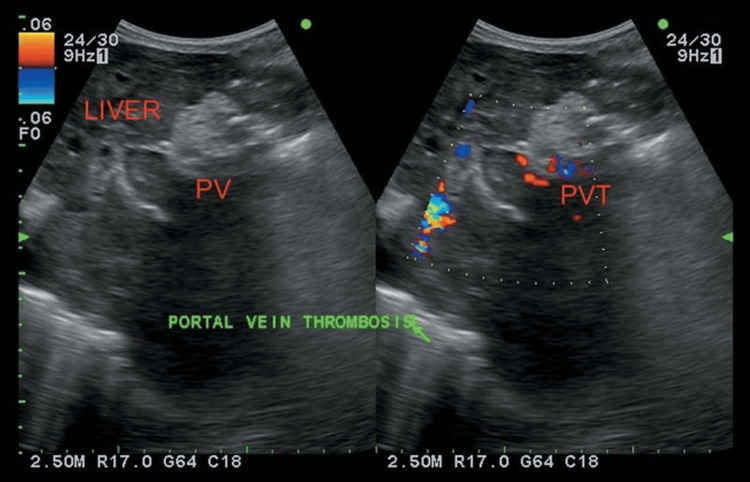
Color Doppler ultrasound demonstrating absence of portal vein flow consistent with portal vein thrombosis.

A percutaneous liver biopsy demonstrated moderately differentiated HCC. Serum alpha-fetoprotein (AFP) level was elevated at >200 ng/mL, with an AFP-L3 fraction of 71.8%. Immunohistochemistry was positive for AFP, glypican-3, HepPar-1, and focal arginase, and negative for cytokeratin 7 (CK7), AE1/AE3, and carcinoembryonic antigen (CEA). A subsequent lung biopsy confirmed metastatic HCC.

During hospitalization, the patient developed progressive respiratory failure in the setting of malignancy-associated hypercoagulability, with extensive thrombosis including bilateral pulmonary emboli, portal vein thrombosis, and inferior vena cava thrombus. Following hematology consultation, anticoagulation was initiated with intravenous heparin under protocol-driven partial thromboplastin time (PTT) monitoring. Due to the need for image-guided lung biopsy, heparin was temporarily held, and the patient was transitioned to weight-based enoxaparin. Ongoing cancer-related weight loss necessitated frequent dose adjustments, with a plan for discharge on enoxaparin.

Empiric antibiotic therapy with ceftriaxone and doxycycline was initiated for suspected community-acquired pneumonia; however, infectious etiologies were ultimately excluded. Despite these interventions, respiratory status continued to decline due to the combined effects of extensive metastatic pulmonary involvement and superimposed thromboembolic disease. Given worsening hepatic function, extensive metastatic disease, and declining performance status, the patient was deemed not to be a candidate for systemic therapy. Endoscopic evaluation for variceal screening was deferred due to high procedural risk, precluding initiation of bevacizumab-based therapy.

The patient was discharged on supplemental oxygen but returned shortly thereafter with worsening hypoxic respiratory failure. Following admission to the intensive care unit and multidisciplinary discussions regarding prognosis, goals-of-care discussions were held, and he was transitioned to comfort measures. His clinical course was rapidly progressive, with a decline from presentation to the end of the clinical course occurring within approximately four to five weeks. He subsequently died from cardiopulmonary arrest in the setting of advanced metastatic HCC.

## Discussion

HCC is an aggressive malignancy with a strong propensity for vascular invasion and extrahepatic dissemination, particularly to the lungs [1,4]. Despite advances in surveillance and systemic therapy, HCC remains a major cause of cancer-related mortality worldwide because many patients present with advanced-stage disease and limited therapeutic options [2,3]. Pulmonary metastases most commonly manifest as multiple discrete nodules; however, less common presentations such as lymphangitic carcinomatosis are associated with rapidly progressive respiratory compromise and poor prognosis [4]. In the present case, diffuse pulmonary metastatic involvement with suspected lymphangitic spread contributed directly to refractory hypoxic respiratory failure and rapid clinical deterioration. A comparison of the clinical features and outcomes of the present case with prior studies of HCC with extrahepatic metastases is summarized in Table 2.

**Table 2:** Comparison of present case with prior studies of hepatocellular carcinoma with extrahepatic metastases. Comparison of clinical features, disease behavior, and outcomes between the present case and prior studies of hepatocellular carcinoma with extrahepatic metastases. IVC: inferior vena cava

Feature	Present Case	Sung et al. (2021) [2]	Forner et al. (2018) [3]	Katyal et al. (2000) [4]
Study type	Single case	Epidemiologic study	Review article	Imaging study
Pulmonary metastases	Diffuse, innumerable nodules	Reported in advanced disease	Reported	Most frequent site
Lymphangitic carcinomatosis	Present	Not emphasized	Not emphasized	Not emphasized
Vascular invasion	Portal vein thrombosis, IVC thrombus	Not specified	Common in advanced disease	Reported
Pulmonary embolism	Present	Not emphasized	Not emphasized	Not emphasized
Clinical course	Rapid (four to five weeks)	High mortality	Poor prognosis	Not specified
Outcome	Death	Reduced survival	Reduced survival	Not specified

Vascular invasion and malignancy-associated hypercoagulability represented additional major drivers of disease progression in this patient. Portal vein thrombosis is a recognized complication of advanced HCC associated with impaired hepatic perfusion, accelerated tumor progression, and reduced survival. Patients with HCC appear particularly susceptible to thrombotic complications because of the combined effects of vascular invasion, cirrhosis-associated hemostatic alterations, and tumor-driven hypercoagulability [5]. In this patient, the coexistence of bilateral pulmonary emboli, portal vein thrombosis, and inferior vena cava thrombus reflected a profound systemic prothrombotic state involving multiple vascular territories.

The thrombotic burden observed in this case also highlights the complex hemostatic alterations characteristic of cirrhosis and HCC. Contemporary understanding of cirrhosis-associated coagulation recognizes a state of “rebalanced hemostasis,” in which simultaneous reductions in procoagulant and anticoagulant factors create a fragile equilibrium predisposing patients to both thrombosis and bleeding [9]. Conventional coagulation studies such as prothrombin time/international normalized ratio (PT/INR) and PTT do not accurately reflect true hemostatic balance and should not be interpreted as evidence of auto-anticoagulation. In patients with HCC, tumor-related mechanisms including increased tissue factor expression, impaired fibrinolysis, and release of procoagulant extracellular vesicles may further shift this equilibrium toward hypercoagulability [9]. In the present case, the combined effects of cirrhosis-associated hemostatic dysregulation and malignancy-driven thrombogenesis likely contributed synergistically to the development of extensive multifocal thrombosis.

Management of cancer-associated thrombosis in advanced HCC remains particularly challenging because treatment decisions require balancing substantial thrombotic risk against significant hemorrhagic potential. Prior studies in high-risk malignancies have demonstrated that thromboprophylaxis may reduce venous thromboembolism without substantially increasing major bleeding in selected patient populations. However, the coexistence of cirrhosis, portal hypertension, suspected varices, and extensive metastatic disease in this patient created a uniquely high-risk clinical scenario in which standard anticoagulation paradigms could not be safely generalized.

The practical delivery of anticoagulation in this case further underscores these therapeutic limitations. Progressive cancer-associated cachexia required repeated adjustments of weight-based enoxaparin dosing, increasing the risk of both subtherapeutic and supratherapeutic anticoagulation. In addition, temporary interruption of heparin for image-guided lung biopsy left the patient without thromboprophylaxis despite active multifocal thrombosis. These challenges were compounded by the unreliability of conventional coagulation studies in cirrhosis, making standard heparin titration protocols less predictable [9]. Collectively, these factors demonstrate that even when anticoagulation is clearly indicated, its safe administration may be substantially compromised in patients with advanced HCC complicated by cirrhosis, cachexia, and concurrent procedural requirements.

The simultaneous presence of diffuse pulmonary metastases, suspected lymphangitic carcinomatosis, and extensive thromboembolic disease produced a synergistic pathophysiologic process culminating in rapidly progressive hypoxic respiratory failure. Pulmonary lymphangitic carcinomatosis results from diffuse infiltration of pulmonary lymphatics by malignant cells, leading to impaired gas exchange, progressive hypoxemia, and diffuse interstitial pulmonary abnormalities that may closely mimic infectious etiologies. In this patient, the initial concern for atypical infection, particularly tuberculosis in the setting of recent travel and systemic symptoms, reflected the diagnostic complexity commonly encountered in these presentations.

Importantly, the prognostic and therapeutic implications of lymphangitic carcinomatosis vary considerably according to primary tumor histology. Previous radiographic studies have demonstrated that some patients with lymphangitic carcinomatosis may experience relatively prolonged clinical courses despite diffuse pulmonary involvement [10]. In breast cancer, lymphangitic carcinomatosis has been associated with symptomatic improvement and prolonged survival following systemic therapy and corticosteroid administration [11]. Similarly, substantial responses have been reported in colorectal carcinoma treated with anti-EGFR therapy [12] and in lung adenocarcinoma treated with targeted therapies directed at actionable mutations [13]. In contrast, therapeutic options for lymphangitic carcinomatosis secondary to HCC remain markedly limited.

Management of advanced HCC in this setting is further constrained by the limitations of currently available systemic therapies. Atezolizumab plus bevacizumab has demonstrated survival benefit and remains first-line therapy for unresectable HCC in eligible patients [6]. However, guideline-based management requires adequate hepatic reserve and prior endoscopic screening for varices because of the bleeding risk associated with bevacizumab therapy [7]. In the present case, progressive hepatic dysfunction, poor functional status, and clinical instability precluded endoscopic evaluation, thereby preventing safe initiation of bevacizumab-based treatment. Even if lymphangitic spread had been the sole pulmonary manifestation in this patient, underlying cirrhosis, portal hypertension, and declining hepatic reserve would still have significantly restricted the safe administration of systemic therapy. This contrast highlights how the biologic behavior of the primary malignancy fundamentally determines prognosis and therapeutic potential when lymphangitic carcinomatosis occurs.

Radiographically, lymphangitic carcinomatosis in this patient was inferred based on diffuse interstitial pulmonary involvement and widespread nodularity on CT imaging, findings consistent with previously described radiologic patterns [10]. Although histopathologic confirmation of lymphatic invasion was not obtained, the imaging findings combined with biopsy-proven metastatic HCC strongly supported the diagnosis.

Overall, this case underscores the complex interplay between metastatic pulmonary disease, vascular invasion, cirrhosis-associated hemostatic dysregulation, and malignancy-driven hypercoagulability in advanced HCC. These overlapping processes contributed to rapid respiratory decline, profound thrombotic complications, and severely limited therapeutic options. The case also highlights the practical barriers encountered in advanced HCC management, including anticoagulation challenges, procedural limitations, and inability to safely initiate guideline-directed systemic therapy. Early recognition of malignant etiologies in patients presenting with diffuse pulmonary infiltrates and systemic symptoms, together with prompt multidisciplinary involvement and timely goals-of-care discussions, remains essential in similarly advanced presentations.

## Conclusions

This case highlights an aggressive presentation of advanced HCC characterized by diffuse pulmonary metastases with suspected lymphangitic carcinomatosis, multifocal malignancy-associated thrombosis, and rapidly progressive acute hypoxic respiratory failure. The simultaneous presence of metastatic pulmonary involvement, vascular invasion, and profound hypercoagulability created a synergistic pathophysiologic process that severely limited therapeutic options and culminated in rapid clinical deterioration. This presentation underscores the importance of maintaining a high index of suspicion for metastatic malignancy and cancer-associated thromboembolism in patients presenting with diffuse bilateral pulmonary opacities, systemic symptoms, and hepatic dysfunction, even when infectious etiologies are initially favored. Early recognition of extrahepatic dissemination, cirrhosis-associated hemostatic dysregulation, and thrombotic complications is essential, as these findings carry major prognostic significance and directly impact diagnostic and therapeutic decision-making.

Additionally, this case emphasizes the practical limitations of managing advanced HCC in the setting of cirrhosis, cachexia, procedural needs, and poor functional status, where standard systemic therapies and anticoagulation strategies may become difficult or unsafe to implement. Early multidisciplinary evaluation, timely assessment of therapeutic candidacy, and proactive goals-of-care discussions remain critical to optimizing patient-centered care in similarly advanced presentations.
